# Toward a Continuous Intravascular Glucose Monitoring System

**DOI:** 10.3390/s110100409

**Published:** 2010-12-31

**Authors:** Brooke Beier, Katherine Musick, Akira Matsumoto, Alyssa Panitch, Eric Nauman, Pedro Irazoqui

**Affiliations:** 1 The Weldon School of Biomedical Engineering, Purdue University, West Lafayette, IN 47907, USA; E-Mails: kmmusick@purdue.edu (K.M.); apanitch@purdue.edu (A.P.); enauman@purdue.edu (E.N.); pip@purdue.edu (P.I.); 2 Department of Bioengineering, The University of Tokyo, 7-3-1 Hongo, Bunkyo-ku, Tokyo 113-8656 Japan; E-Mail: amatsumoto@bmw.t.u-tokyo.ac.jp (A.M.); 3 Department of Basic Medical Sciences, Purdue University, West Lafayette, IN 47907, USA; 4 School of Mechanical Engineering, Purdue University, West Lafayette, IN 47907, USA

**Keywords:** glucose monitoring, hydrogels, biosensors, polymers, continuous, intravascular, stent, wireless

## Abstract

Proof-of-concept studies that display the potential of using a glucose-sensitive hydrogel as a continuous glucose sensor are presented. The swelling ratio, porosity, and diffusivity of the hydrogel increased with glucose concentration. In glucose solutions of 50, 100, 200, and 300 mg/dL, the hydrogel swelling ratios were 4.9, 12.3, 15.9, and 21.7, respectively, and the swelling was reversible. The impedance across the hydrogel depended solely on the thickness and had an average increase of 47 Ω/mm. The hydrogels exposed to a hyperglycemic solution were more porous than the hydrogels exposed to a normal glycemic solution. The diffusivity of 390 Da MW fluorescein isothiocyanate in hydrogels exposed to normal and hyperglycemic solutions was examined using fluorescence recovery after photobleaching and was found to be 9.3 × 10^−14^ and 41.4 × 10^−14^ m^2^/s, respectively, compared to 6.2 × 10^−10^ m^2^/s in glucose solution. There was no significant difference between the permeability of hydrogels in normal and hyperglycemic glucose solutions with averages being 5.26 × 10^−17^ m^2^ and 5.80 × 10^−17^ m^2^, respectively, which resembles 2–4% agarose gels. A prototype design is presented for continuous intravascular glucose monitoring by attaching a glucose sensor to an FDA-approved stent.

## 1. Introduction

Diabetes is one of the leading causes of death by disease, killing approximately 3 million people around the world and 180,000 Americans each year [1]. Approximately 171 million people worldwide are currently diagnosed with diabetes and the estimated global prevalence is predicted to be 366 million in 2030 [2]. The number of diagnosed cases has risen by 11% over the last 5 years [3] and is expected to double within the next 25 years due to factors such as population growth, increased life expectancy, increased prevalence of obesity, and physical inactivity [4]. In the United States diabetes costs the healthcare system upwards of $174 billion annually and this number is on the rise [5].

Diabetes is a manageable disease; however management requires accurate and frequent measurements of a patient’s glucose levels to ensure that they stay within the normal range of 80 to 110 mg/dL to avoid hypoglycemic and hyperglycemic episodes [6]. Abnormal glucose levels can cause blood vessel damage, which in the long-term may result in adult blindness, serious kidney disease, the need for amputations as a result of neuropathy, and a 2–4× increase in the incidence of heart failure [3]. The Diabetes Control and Complications Trial (DCCT) found that tight control of glucose levels decreases the occurrence and severity of long-term complications [7].

In order to achieve the benefits of tight control, new technology to continuously monitor blood glucose levels is being developed with the aim to minimize the pain and effort on the part of the patient. There are a few limitations with current continuous glucose monitors: (1) they measure blood glucose levels indirectly from the interstitial fluid which lag behind blood glucose levels (2) they can only be implanted for short periods of time (3) they are expensive and (4) they are not as accurate as blood glucose sensors [8]. To overcome a few of these limitations, we propose to monitor intravascular glucose levels continuously using a glucose-sensitive hydrogel embedded in an FDA-approved stent. A stent would provide the sensor with constant access to the bloodstream, the mechanical support, and a means to transmit data while the stent maintains vessel patency [9]. Importantly, a link has been established between diabetes and heart failure, as the prevalence of diabetes in heart failure populations is close to 20% compared to 4–6% in control populations, thus justifying this combined technology [10]. Further, cardiovascular disease has surpassed diabetic neuropathy as the leading cause of early death in juvenile type I diabetics [11].

Recent research in the field of continuous glucose monitoring has focused on developing glucose-sensitive hydrogels. To transduce signal, the sensor designs exploit hydrogels swelling in response to chemical stimuli [12]. In this work, we explore and characterize a poly(*N*-isopropylacrylamide) (PNIPAAm)-based glucose-sensitive hydrogel developed by Kataoka *et al.* that also contains a phenylboronic acid derivative, which is known to form complexes with polyol compounds, like glucose, instead of proteins or enzymes [13,14]. We hypothesized that the hydrogel swelling would correlate to measureable impedance values that would correlate to physiological glucose concentrations. To validate this we investigated mass transport properties and pore size within the hydrogel as well as impedance values after hydrogel swelling. The data suggests that the hydrogel holds promise for use as a transducer for continuous glucose monitoring.

## 2. Experimental Section

### 2.1. Hydrogel Synthesis

Hydrogel was synthesized with an AAPBA (= 3-acrylamidophenylboronic acid) content of 10 mol % as follows: Under argon atmosphere, N-isopropylacrylamide (3.37 g), 3-acrylamidophenylboronic acid (0.63 g), and N,N‘-methylene-bis-acrylamide (0.05 g) as a cross-linker were dissolved in 20 mL of DMSO (dimethyl sulfoxide) with initiator 2,2‘-azobis(2,4 dimethylvaleronitrile) (1 mg/mL). The prepared solution was injected between two Teflon sheets (10 cm × 10 cm) separated by a Teflon gasket (1.0 mm thickness) and backed by glass plates and polymerized at 60 °C for 16 h. The formed hydrogel slab was immersed successively into a series of DMSO/water mixtures (100/0, 75/25, 50/50, 25/75, and 0/100) to remove unreacted compounds. The slab was kept at least one day in each solution [13].

### 2.2. Preparation of Glucose Solutions

Normal physiological blood glucose levels range between 80 and 110 mg/dL and those at or below 70 mg/dL are hypoglycemic and those at or above 180 mg/dL are hyperglycemic [3,6]. Glucose solutions were prepared to model normal, hyperglycemic, and hypoglycemic conditions. A 50 mM 2-(Cyclohexylamino)ethanesulfonic acid, 100 mM NaCl stock solution (pH = 9) was prepared and α-D-Glucose was added to various amounts of the stock solution to make glucose solutions of 0, 50, 100, 200, 300, and 500 mg/dL. The concentrations of the solutions were verified with a Red Glucose/Glucose Oxidase Assay Kit.

### 2.3. Swelling Ratio Studies

Tests were performed to characterize and quantify the volume change, both shrinking and swelling, of the hydrogel as a function of glucose concentration and time. The swelling ratios were calculated using,

(1) $$\mathrm{Swelling Ratio}=\frac{{\mathrm{Weight}}_{\mathrm{time}}}{{\mathrm{Weight}}_{\mathrm{initial}}}$$

where *Weight_time_* is the weight of the hydrogel at the time of measurement and *Weight_initial_* is the initial weight of the hydrogel (prior to immersion in a solution with a glucose concentration).

#### 2.3.1. Swelling Study: Swelling Ratio *versus* Time

To initialize the hydrogel, a slab was placed in a 0 mg/dL glucose solution for 1 week at 25 °C. Following initialization, the hydrogel was sliced into 12 equal size rectangular pieces (42.5 ± 7.05 mg). Each sample was blotted dry, weighed, and assigned to a vial containing 5 mL of a 50, 100, 200, or 300 mg/dL glucose solution. The samples were randomly assigned to each of the four concentrations. Each sample was monitored over one month and 25 times each sample was taken out of its respective vial, blotted dry, weighed, and returned to its respective vial. As the swelling ratios reached equilibrium, the time between measurements began to increase as less change was observed. On average prior to the samples reaching equilibrium the time between measurements was 16 h and after the sample reached equilibrium this time was increased on average to 59 h.

#### 2.3.2. Shrinking Study: Swelling Ratio *versus* Time

To assess the reversibility of hydrogel swelling, an experiment was performed in which hydrogels that had reached their equilibrium swelling ratios at a certain glucose concentration were transferred to a 100 mg/dL glucose solution. Specifically, 12 hydrogels (396.63 ± 187.90 mg) that had reached an equilibrium swelling ratio in a glucose solution of 50 (n = 3), 100 (n = 3), 200 (n = 3), or 300 (n = 3) mg/dL were blotted dry, weighed, and placed in 12 mL of a 100 mg/dL glucose solution. Over the course of one month, 11 times the hydrogels were removed from their vials, blotted dry, weighed, and returned to their respective vial. This process was performed daily for the first four days and performed less frequently as less change was apparent between measurements.

### 2.4. Impedance Measurements

To demonstrate the feasibility of this hydrogel as a glucose sensor, the impedance through the hydrogel was measured in samples exposed to 300 (n = 4) and 500 (n = 4) mg/dL glucose solutions. Two probes connected to a LCR meter were placed in the hydrogels repeatedly at increasing inter-probe distances. For each distance, the magnitude and phase of the impedance was measured.

### 2.5. Scanning Electron Microscopy

In order to examine the microstructure of the hydrogel, scanning electron microscopy (SEM) was performed on three samples exposed to glucose concentrations of 0, 100, and 300 mg/dL. A piece of the initialized hydrogel was sliced into 3 equal size pieces (52.6 ± 2.3 mg). One piece was placed in a vial containing a solution with a 0 mg/dL glucose concentration, another in 100 mg/dL glucose concentration, and a third in 300 mg/dL glucose concentration. The samples were allowed to reach their equilibrium swelling ratio over the course of three weeks. Once no change in swelling ratio was observed, samples of each of the hydrogels were sliced from the specimen. Each sample was placed into a slit holder using cryo adhesive to anchor. The sample holder was plunged into liquid nitrogen slush. A vacuum was pulled and the sample was transferred to the Gata Alto 2,500 pre-chamber (cooled to approximately −170 °C). After fracturing the sample with a cooled scalpel to produce a free-break surface, the sample was sublimated at −85 °C for 30 min followed by sputter coating for 120 s with platinum. The sample was then transferred to the microscope cryo-stage (maintained at −130 °C) for imaging. The porosity ratios of the hydrogels were calculated using,

(2) $$\mathrm{Porosity Ratio}=\frac{\mathrm{Void Space}}{\mathrm{Total Space}}$$

where *Void Space* (pixels) was defined as the porous area of the hydrogel and the *Total Space* (pixels) was defined as the total area of the analyzed specimen.

### 2.6. Diffusivity Measurements

The diffusivity of 390 Da MW fluorescein isothiocyanate (FITC) through glucose solution and hydrogels that had reached equilibrium swelling upon exposure to 100 and 300 mg/dL glucose solutions was investigated using FRAP. A vibratome was used to slice hydrogel samples with thicknesses of approximately 250 μm. Non-conjugated FITC molecules were added to the 100 and 300 mg/dL glucose solutions in which the hydrogel samples were immersed 48 h prior to the start of the experiment. The fluorescent samples were viewed with a FluoView 1,000 confocal system connected to a TE2000 inverted microscope. The specimens were scanned at a low laser intensity of 5% to locate an area of approximate uniform fluorescence. A circular bleaching area (radius = 30 μm) was selected and the experiment was conducted with a 488 nm laser and a bleaching intensity of 100% at a 60× magnification. Following bleaching, the fluorescent response in the field of view was monitored over time in 23 concentric circles with radii ranging from 4.14 to 62.3 μm for the hydrogel samples and 12.28 to 186.9 μm for the glucose solution. Following bleaching, the average pixel intensity of each region of interest (ROI) at various time points was normalized using,

(3) $$\bar{I}{\left(t,r_{i}\right)}_{\mathrm{Normalized}}=\frac{\bar{I}\left(t,r_{i}\right)-\bar{I}\left(t_{0},r_{0}\right)}{\bar{I}\left(t,r_{\infty }\right)-\bar{I}\left(t_{0},r_{0}\right)}$$

(defined in 1976 by Axelrod *et al.*) such that the average intensity within the bleached ROI ranged between 0 and 1 [15]. In this equation, *I̅*(*t*, *r_i_*)*_Normalized_* is the normalized average intensity of the i^th^ ROI at time t, *I̅*(*t*, *r_i_*) is the average true intensity of the i^th^ ROI at time t, *I̅*(*t*_0_, *r*_0_) is the intensity of the center of the bleached area of the sample at time 0, which is defined as the time of bleaching, and *I̅*(*t*, *r*_∞_) is the intensity of the infinite reservoir diffusing into the bleached area at time t.

A COMSOL transient diffusion model was developed using initial intensity conditions from each ROI. For each time point, the initial concentrations observed in each ROI for the hydrogels exposed to 100 (n = 13) and 300 (n = 7) mg/dL were averaged for each of the samples and modeled in COMSOL. This was repeated for the initial data obtained from the glucose solution. Different diffusion coefficients were applied to the models and the intensity profiles over each ROI as a function of time were simulated. For each diffusion coefficient, the COMSOL model data was compared to the experimental data and the model that minimized the sum of squared differences between the experimental and model data was determined to be the best-fit model. Assumptions applied to the FRAP method include: Fickian diffusion is the only mode of mass transport, diffusion only occurs in the radial direction, the FITC does not interact with the hydrogel, the photobleaching is an irreversible process, and the bleaching process does not alter the structure of the hydrogel.

In order to quantify the difference between the diffusivity of the 390 Da MW FITC in glucose solution and in hydrogels exposed to the 100 and 300 mg/dL glucose solution, the hindrance ratio was calculated using,

(4) $$\mathrm{Hindrance Ratio}=\frac{D_{\mathrm{FITC, GLUCOSE SOLUTION}}}{D_{\mathrm{FITC, HYDROGEL}}}$$

where *D*_*FITC,GLUCOSE SOLUTION*_ (m^2^/s) is the diffusion coefficient for the FITC in the glucose solution, and *D*_*FITC,HYDROGEL*_ (m^2^/s) is the diffusion coefficient for the FITC in the hydrogel.

### 2.7. Permeability Measurements

A sample of the hydrogel was initialized in a 0 mg/dL glucose solution for one week and then sliced into nine pieces of equal size (26.53 ± 4.4 mg). Each piece was blotted dry, weighed, measured, and placed into a 4-mm diameter cylindrical tube. Six of the tubes were filled with 300 mg/dL glucose solution, and the other three were filled with 100 mg/dL glucose solution to initiate hydrogel swelling. The ends of the tubes were covered with parafilm to eliminate evaporation and leaking, and the hydrogels were allowed to swell and form a tight seal. Permeability columns were constructed using 1/4”-inner-diameter Tygon tubing attached vertically to a support and each hydrogel sample was connected with a liquid tight seal to the bottom of a column. The columns were filled with glucose solutions that matched those that the hydrogels were initially exposed to and the initial height of the solution column and time were recorded. Over time, multiple measurements of the height of the column above the hydrogel were recorded. From these, the flow rate of the liquid Q (m^3^/s) was obtained and divided by the cross-sectional area of the specimen *A_m_* (m^2^) to obtain the velocity *v* (m/s) of the liquid. Darcy’s Law was used to calculate the permeability constant for each of the samples,

(5) $$v=\frac{Q}{A}=\frac{\kappa }{\mu }\left(\frac{\Delta P}{L_{m}}\right)$$

where *κ* is Darcy permeability (m^2^), *L_m_* (m) is the length of the specimen, *P* (Pa) is pressure, and *μ* (Pa·s) is the dynamic viscosity of the fluid.

## 3. Results

### 3.1. Swelling Ratio

**Figure 1.**
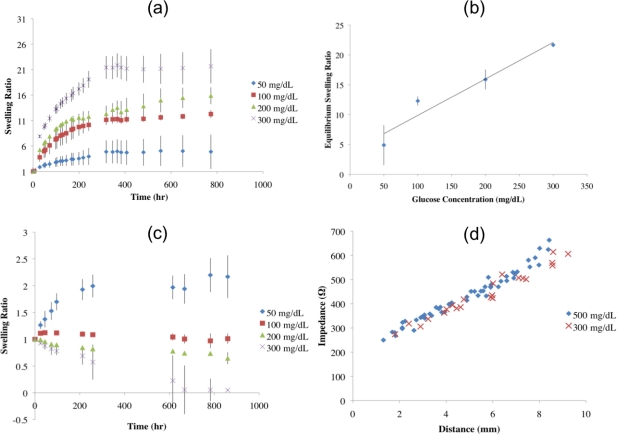
**(a)** Swelling ratio *versus* time for hydrogel samples that were exposed to glucose solutions of 50, 100, 200, and 300 mg/dL after initially being placed in 0 mg/dL glucose solution. Equilibrium was reached after approximately 300 h. **(b)** Equilibrium swelling ratio of the hydrogels *versus* glucose concentration of exposure. **(c)** Swelling ratio *versus* time of hydrogels that had reached equilibrium swelling ratios in 50, 100, 200, and 300 mg/dL glucose solutions and then transferred to a glucose solution with a glucose concentration of 100 mg/dL. **(d)** Impedance *versus* distance through hydrogels that were exposed to 300 and 500 mg/dL glucose solutions.

A plot of the average swelling ratio *versus* time for each glucose concentration group is shown in Figure 1(a). The hydrogels displayed a logarithmic swelling trend over time when transferred from a 0 mg/dL glucose solution to one containing 50, 100, 200, or 300 mg/dL glucose. As shown in Table 1, increased degree of hydrogel swelling correlated with increasing glucose concentration. Specifically, hydrogels that were transferred from a 0 mg/dL glucose solution to a 50 mg/dL glucose solution had the slowest rate of swelling, followed by the hydrogels exposed to 100 mg/dL glucose, then those exposed to 200 mg/dL glucose; and finally, the hydrogels exposed to the 300 mg/dL glucose solution showed the fastest swelling. The swelling ratios appeared to reach steady state values after approximately 300 h of exposure to the glucose solution. Once the hydrogels reached equilibrium, the percent polymer was calculated by dividing the initial weight of the polymer by the weight of the equilibrated sample; the percent polymer was inversely proportional to the final glucose concentration as shown in Table 1. ANOVA (α = 0.05) revealed that there was a statistically significant difference between the equilibrium swelling ratios of the hydrogels exposed to 50, 100, 200, and 300 mg/dL glucose concentrations (p-value < 0.001). Post hoc analysis using Tukey’s Mean Comparison Test (α = 0.05) revealed that the equilibrium swelling ratio of the hydrogels exposed to a 50 mg/dL glucose solution was significantly different than the equilibrium swelling ratios of the hydrogels exposed to 100, 200, and 300 mg/dL glucose solutions. It was also determined that the equilibrium swelling ratios of the hydrogels exposed to 100 and 200 mg/dL glucose solutions were not statistically different from one another, however the equilibrium swelling ratio of the hydrogels exposed to 300 mg/dL glucose solution showed a statistically significant difference from all other groups.

Figure 1(b) shows the average equilibrium swelling ratio *versus* glucose concentration. Linear regression was performed for average equilibrium swelling ratio on glucose concentration of exposure. For every mg/dL increase in glucose concentration, there was a resultant 6.0% increase in the equilibrium swelling ratio. The coefficient of determination was found to be 0.93. When forcing the regression to fit the swelling ratio to 1.0 at a glucose concentration of 0 mg/dL, the coefficient of determination was reduced to 0.88 and the equilibrium swelling ratio was found to increase by 7.4% for every mg/dL increase in glucose concentration.

Overall, when the hydrogels were transferred from one glucose concentration to 100 mg/dL they behaved as expected. The group initially exposed to a solution of 100 mg/dL glucose served as a control. The swelling ratio for each sample and the average swelling ratio for each concentration group are plotted in Figure 1(c). The samples that had equilibrated in a 50 mg/dL glucose solution swelled significantly when placed in the 100 mg/dL glucose solution, whereas the samples that had equilibrated in a glucose solution of 200 and 300 mg/dL significantly shank when transferred to the 100 mg/dL glucose solution. As expected, the samples that had already equilibrated to a 100 mg/dL glucose solution did not fluctuate in mass substantially when transferred to the new 100 mg/dL glucose solution. Two hydrogel samples, one from the 50 mg/dL glucose solution group and one from the 300 mg/dL glucose solution group were excluded from the data analysis due to degradation caused by the extensive handling required by this study. ANOVA (α = 0.05) revealed there was a statistical difference between the equilibrium swelling ratios of the hydrogels that were transferred to 100 mg/dL glucose solution following equilibration in glucose solutions of 50, 100, 200, and 300 mg/dL (p-value = 0.0114).

### 3.2. Impedance

For all values, the phase angle remained between −3 and −8°, indicating a largely resistive impedance. As shown in Figure 1(d), for the hydrogels exposed to 300 and 500 mg/dL glucose solutions, the magnitude of the impedance increased linearly with distance with an average slope of 47 Ω/mm (R^2^ = 0.95). The average slope of the best-fit lines for impedance *versus* distance for hydrogels exposed to 300 and 500 mg/dL glucose solutions were 44 Ω/mm (R^2^ = 0.96) and 49 Ω/mm (R^2^ = 0.97), respectively.

### 3.3. Scanning Electron Microscopy

**Figure 2.**
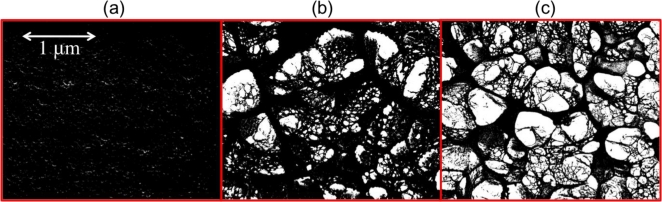
SEM images converted to black (hydrogel) and white (pore) scale (50000×) of the hydrogels in various glucose concentrations. **(a)** 0 mg/dL, **(b)** 100 mg/dL, **(c)** 300 mg/dL.

SEM images were imported into Image-Pro Plus 5.1 to compare the porosity of the hydrogels that were exposed to 0, 100, and 300 mg/dL glucose solutions (Figure 2). The ratio of the void space to the total sample area was determined for the hydrogels exposed to 0 (n = 10), 100 (n = 10), and 300 (n = 18) mg/dL glucose solutions. As expected, as glucose concentration increased, pore size increased as shown in Table 2; the hydrogels that were exposed to a solution containing 0 mg/dL glucose did not have visible pores, therefore for these samples pore size was assumed to be approximately zero. An unpaired t-test (α = 0.05) analysis of the SEM images revealed that the ratio of the void space to total hydrogel sample area for the hydrogels exposed to 100 and 300 mg/dL glucose solutions were statistically significantly different from one another (p-value < 0.0001). The 95% confidence intervals for the porosity ratios for the hydrogels exposed to 100 and 300 mg/dL glucose solutions were (0.2604, 0.3085) and (0.3511, 0.3918), respectively.

### 3.4. Diffusivity

**Figure 3.**
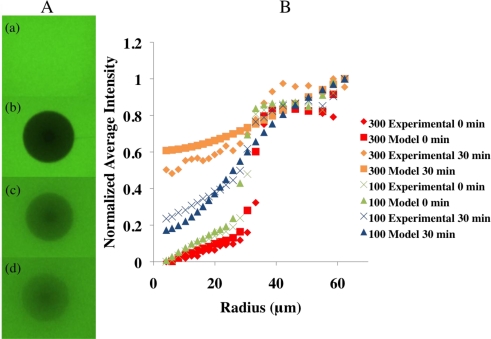
**(A)** (a) Initial fluorescent sample. (b) Sample immediately following bleaching. (c) Sample 15 min after bleaching. (d) Sample 30 min after bleaching. **(B)** Normalized average intensity *versus* radius for experimental and model data of hydrogels exposed to 100 and 300 mg/dL glucose solution at time points of 0 and 30 minutes following photobleaching. The hydrogel exposed to the 300 mg/dL glucose solution exhibited faster recovery after 30 min following photobleaching than the hydrogel exposed to 100 mg/dL glucose solution.

To demonstrate the FRAP procedure, images were taken of a sample of hydrogel before photobleaching, immediately after photobleaching, and 15 min and 30 min after photobleaching [Figure 3(A)]. The diffusion coefficients for the FITC in the hydrogels exposed to 100 and 300 mg/dL glucose solutions were determined and the results are summarized in Table 2 and Figure 3(B). An unpaired t-test (α = 0.05) analysis of the diffusion coefficients for FITC through the hydrogels exposed to 100 and 300 mg/dL glucose solution showed they were statistically significantly different (p-value = 0.0003). The 95% confidence intervals for the diffusion coefficients for the hydrogels exposed to 100 and 300 mg/dL glucose solutions are (6.19 × 10^−14^ m^2^/s, 1.24 × 10^−13^ m^2^/s) and (1.79 × 10^−13^ m^2^/s, 6.50 × 10^−13^ m^2^/s), respectively. The effectiveness of the numerical model was also demonstrated in Figure 3(B). The average diffusivity of FITC in free solution was found to be 6.2 × 10^−10^ m^2^/s (standard deviation=1.3 × 10^−10^ m^2^/s), which is similar to published values for glucose in water (6.0 × 10^−10^ m^2^/s), thus validating the method [16]. The diffusivity of the FITC in the hydrogel exposed to 300 mg/dL glucose solution was approximately 4 orders of magnitude slower than the diffusivity of the FITC in glucose solution. The hindrance ratios for the hydrogels exposed to the 100 and 300 mg/dL glucose solutions were found to be 6.7 × 10^3^ and 1.5 × 10^3^, respectively.

### 3.5. Permeability

Using Equation 5 the average permeability of the samples exposed to 100 and 300 mg/dL glucose solution over ten days was determined (Table 2). An unpaired t-test (α = 0.05) analysis revealed no statistical difference (p-value = 0.5235) between the permeability of the hydrogels exposed to 100 and 300 mg/dL glucose solutions. The 95% confidence interval for the permeability of the hydrogels exposed to the 100 and 300 mg/dL glucose solution are (1.68 × 10^−17^ m^2^, 8.83 × 10^−17^ m^2^) and (4.74 × 10^−17^ m^2^, 6.87 × 10^−17^ m^2^), respectively. The uncertainty in the calculation is 3.27 × 10^−18^ m^2^. The permeability was measured over a pressure range of 8,000 to 9,000 Pa.

## 4. Discussion

This preliminary characterization demonstrates the potential for using a glucose-sensitive hydrogel as the basis for an *in vivo*, continuous glucose sensor. The ability to relate glucose concentration to the volume and impedance change of the hydrogel was demonstrated. There is a direct relationship between the glucose concentration to which the hydrogel was exposed and the equilibrium swelling ratio and swelling rate of the hydrogel. It was found that higher glucose concentrations resulted in higher equilibrium swelling ratios and faster swelling rates. Statistical analysis using ANOVA (α = 0.05) and a post-hoc Tukey’s Mean Comparison Test (α = 0.05) revealed that all equilibrium swelling ratio groups were statistically significantly different from one another except the hydrogels exposed to 100 and 200 mg/dL glucose solutions. The results suggest that with optimization of hydrogel dimensions and chemistry to increase the hydrogel’s sensitivity and response time, it is feasible that this hydrogel could be used as a main component of a glucose-sensing device that uses Micro-Electro-Mechanical Systems (MEMS) technology.

For this monitoring application, it is critical that the hydrogel swelling responds to changing glucose concentrations. This behavior was examined in the second study, and ANOVA (α = 0.05) revealed there was a statistical difference between the equilibrium swelling ratios of the hydrogels that were transferred to 100 mg/dL glucose solution following equilibration in glucose solutions of 50, 100, 200, and 300 mg/dL. Therefore, the swelling process is reversible and the binding and unbinding of glucose from the sensing moiety is effective and quantifiable.

In addition to glucose concentration, the gel volume is dependent on temperature and ionic strength, and these factors can vary throughout the course of a day or from day to day. For example, core body temperature can vary by 1 °C [17] and normal sodium levels range from 135–147 mmol/L [18]. To compensate for these cross-sensitivities, we propose collecting data points from two sets of electrodes. The gel between the first set of electrodes will be glucose-sensitive. The gel between the second set of electrodes will be identical in every way to the first, with the exception that it will lack sensitivity to glucose. Thus, the two measurements can be calibrated so as to extract the volume change due to glucose alone.

Some notable limitations were present due to the nature of the swelling ratio studies. First, multiple weight measurements were required, which resulted in the frequent handling of the hydrogels. Despite using extreme caution, small bits of the hydrogel were likely lost over time, and second, inconsistent manual drying prior to each weighing may also have contributed to the deviations in swelling ratio.

A practical limitation was observed in the time it takes the hydrogels to reach their equilibrium swelling ratio. The target response time for continuous glucose monitors is approximately 5 min to facilitate timely insulin administration [8]. Upon exposure to a higher glucose concentration, the hydrogel took approximately 300 h to reach its equilibrium swelling ratio and upon exposure to a lower glucose concentration, the hydrogel took almost 600 h to reach its equilibrium swelling ratio. The fact that the shrinking of the hydrogel took longer than the swelling was also observed by Matsumoto *et al.* [19]. However, it is important to note that samples with large dimensions, on the order of centimeters, were used in this study in order to increase the accuracy of the weight measurements. In a clinical application of the hydrogel as a glucose sensor, the size of the hydrogel would be on the order of micrometers. It is hypothesized that samples with smaller dimensions would reach their equilibrium swelling ratio quicker than the larger hydrogels in this study. In particular, diffusive length scales are governed by the Fourier Number for mass transport, $F_{0}=\frac{\mathrm{Dt}}{L^{2}}$, where *D* is the diffusivity, *t* is the characteristic timescale, and *L* is the largest diffusive length. Based on the experimental data described herein, dimensional analysis indicates that a sample of the hydrogel whose largest dimension was no more than 170 μm would reach its equilibrium swelling ratio within the proscribed five minutes. The kinetics of the volume changes of hydrogels in general are governed by diffusion-limited transport of the glucose solution through the polymeric network, the completion time for which is inversely proportional to the square of the smallest dimension of the hydrogel [20]. The presented results warrant future optimization of the hydrogel’s dimensions to ensure a timely response for use in sensors.

In order to acquire a quantifiable electrical signal that responds to the change in volume of the hydrogel, the impedances through the hydrogels exposed to 300 and 500 mg/dL glucose solutions were measured. An insignificant difference between the impedance *versus* distance plots was observed in the hydrogels exposed to 300 and 500 mg/dL glucose solutions. It was concluded that the impedance of the hydrogel does not depend on the glucose concentration of exposure; it depends solely on the thickness of the hydrogel sample. From the swelling ratio study, it was determined that the hydrogel is less than 10% polymer if it is exposed to a glucose concentration above 100 mg/dL and the impedance was also independent of the small changes in polymer density with swelling. Thus, the impedance is solely dependant on the ions in the solution that diffuse and permeate through the hydrogel. In an *in vivo* setting, the sensor will function similarly by measuring the impedance across a volume of ionic fluid, with the interprobe distance defined by a hydrogel that has swollen with respect to the surrounding glucose concentration. In future designs, the impedance of the blood will need to be taken into account when examining the impedance of the hydrogel due to swelling as a result of glucose. This is because the impedance of the blood may vary depending on the fluctuation in the ionic concentration. In order to compensate for this in a future design, an additional calibration component may be added that would solely measure the impedance of the surrounding medium.

Determining the mass transfer properties of the hydrogel is essential in order to be able to model the hydrogel’s performance as a sensor, thus we investigated the porous structure of the hydrogel, the diffusivity of a small molecule within the hydrogel as a function of glucose concentration, and the permeability of the hydrogel in a normal and pathological glucose solution. Only two glucose concentrations were examined due to the fact that a clear trend was apparent between the hydrogels exposed to the 100 and 300 mg/dL glucose solution, as shown in Table 2. For these experiments an unbalanced design was used, as data was not excluded from additional experiments performed with some concentration groups. This fact has been taken into account into the statistical analysis with the unpaired t-test (α = 0.05).

Analysis of the SEM images revealed that there was a statistically significant difference between the ratio of the void space to total hydrogel sample area for the hydrogels exposed to 100 and 300 mg/dL glucose solutions. Upon examination of the images it is evident that the hydrogels exposed to the 300 mg/dL glucose solution had more void space when compared to the hydrogels exposed to the 100 mg/dL glucose solutions, as shown in Table 2. This significant difference was expected based on the swelling ratio study previously described, in which the hydrogels exposed to the 300 mg/dL glucose solutions had a lower polymer percentage than the hydrogels exposed to the 100 mg/dL glucose solutions.

To estimate the diffusivity of glucose (molecular weight 180 g/mol) within the hydrogel after reaching an equilibrium swelling ratio in glucose solutions of 100 and 300 mg/dL the fluorophore non-conjugated FITC with a molecular weight of 390 g/mol was used. It was found that the diffusion coefficient of the FITC through the hydrogels exposed to 300 mg/dL glucose solution was larger than the diffusion coefficient of the FITC through the hydrogels exposed to 100 mg/dL glucose solution by a factor of 4.45. This result was hypothesized as the hydrogels exposed to 100 mg/dL glucose solutions have a smaller porosity ratio than the 300 mg/dL. An unpaired t-test analysis of the diffusion coefficients for FITC through the hydrogels exposed to 100 and 300 mg/dL glucose solution showed the difference was statistically significant. Furthermore, the diffusion coefficient of the FITC in 300 mg/dL glucose solution, 6.2 × 10^−10^ m^2^/s, was remarkably similar to what has been found for glucose in water 6.0 × 10^−10^ m^2^/s [16]. This finding not only showed that the diffusivity of the FITC in the hydrogels exposed to 100 and 300 mg/dL glucose solutions was 3 to 4 orders of magnitude slower than the diffusivity of the FITC in glucose solution, it also added confidence to the validity of the experimental method. The hindrance ratios showed that the presence of the hydrogels slowed the diffusion of the FITC molecules by over three orders of magnitude compared to the diffusion in glucose solution.

Other studies in the literature have used the permeability constant to infer information about the microstructure of gels and a sense of the order of magnitude of the permeability of the glucose-sensitive hydrogel can be gathered upon comparison to these findings (Table 3). The hydrogels exposed to 100 and 300 mg/dL glucose solutions were found to have average permeabilities of 5.26 × 10^−17^ m^2^ and 5.8 × 10^−17^ m^2^, respectively, which were found to be similar to the published permeability values of 2, 4, and 7.5% agarose gels [21,22]. Furthermore, an unpaired t-test (α = 0.05) analysis revealed no statistical difference between the permeabilities of the hydrogels exposed to 100 and 300 mg/dL glucose solutions.

The pressures the hydrogels were exposed to in the permeability columns ranged from 8,000 to 9,000 Pa (60–67.5 mmHg). As a comparison, the mean arterial pressure of a normal individual is 83 mmHg and the blood pressure is highest (110 mmHg) in the aorta during systole and is the lowest (70 mmHg) during diastole [23]. Furthermore, the mean pressures observed in the pulmonary artery and right atrium in normal individuals are 17 ± 2 and 6 ± 5 mmHg, respectively [24,25]. The pressures that the hydrogel were exposed to were slightly lower than that of normal mean arterial pressures, but higher than those observed in the rest of the circulatory system.

Future mathematical modeling will be performed using the diffusivity and permeability values found to test numerous designs of the hydrogel to determine which allows for the fastest diffusion of glucose throughout the entire hydrogel, and therefore the quickest response time. To take the modeling one step further, the sensor will be modeled in the bloodstream and attached to a stent.

## 5. Conclusions

Incremental advances in diabetes research have made a long-term, continuous glucose monitoring system feasible within the near future. We aim to develop a long-term, continuous, intravascular glucose monitoring system that will minimize complications due to missed glucose measurements and aid in the management of the disease. The ideal intravascular continuous glucose sensor would last for the lifetime of the patient and have a response time and accuracy equal to the *ex vivo* glucose monitoring systems (<5 seconds and an accuracy in accordance to ISO 15197 which states blood glucose meters must provide results that are within 20% of a laboratory standard 95% of the time)[27]. Subcutaneous glucose sensors have a response time of 5–20 minutes due to the lag between the blood and interstitial glucose levels [8], their accuracy is of great debate, and the sensor lifetime has been found to be only a week due to encapsulation and sensor drift [28,29]. Much work needs to be performed to bring our future glucose sensor to these performance standards. However, these preliminary results show this may be possible in the future, thus providing a superior technology to continuous subcutaneous glucose sensors. Preliminary characterization of the glucose-sensitive hydrogel has been performed and the results will be used to model an optimal glucose sensor design. It can be concluded that the swelling ratio and rate of the hydrogel are directly related to the glucose concentration of exposure. Furthermore, the swelling behavior was determined to be reversible. From this swelling behavior, the impedance of the hydrogel can be measured as a function of hydrogel thickness, however no dependence on glucose concentration of exposure was observed. It was found that the porosity and diffusivity of the hydrogel increase with an increase in the glucose concentration of exposure, however an insignificant difference in the permeability of the hydrogel between exposures to 100 and 300 mg/dL glucose solutions was observed. Based on these experiments, it seems feasible that a continuous, intravascular glucose sensor could be developed with the underlying concept that the binding of glucose with the hydrogel results in a volume change, which results in a measurable change in impedance that is correlated to glucose concentration. We have gathered preliminary data to move forward to optimize and build a prototype of a glucose sensor with this hydrogel and integrate it with our previous work of using FDA-approved stents as antennas for wireless data transfer from within the body [9,30–32].

## Figures and Tables

**Table 1.** Polymer percentage and best-fit trend lines for the swelling ratio *versus* time for hydrogels exposed to 50, 100, 200, and 300 mg/dL glucose solutions.

**Glucose Concentration (mg/dL)**	**Average Equilibrium Swelling Ratio**	**Best-Fit Equation**	**Coefficient of Determination (R^2^)**	**Polymer Percentage**
50	4.88	SR = 0.5307ln(GC) + 1	0.73	32.2 ± 27.8
100	12.28	SR = 1.5695ln(GC) + 1	0.82	8.2 ± 0.5
200	15.88	SR = 1.9614ln(GC) + 1	0.84	6.3 ± 0.6
300	21.66	SR = 3.0425ln(GC) + 1	0.83	4.6 ± 0.1

GC: Glucose concentration of exposure

SR: Equilibrium swelling ratio.

**Table 2.** Porosity ratio, diffusion coefficient, permeability of hydrogels exposed to 100 and 300 mg/dL glucose solution.

**Glucose Concentration (mg/dL)**	**Porosity Ratio**			**Diffusion Coefficient**			**Permeability**		
	**n**	**Average**	**CV**	**n**	**Average × 10^−14^ m^2^/s**	**CV**	**n**	**Average × 10^−17^ m^2^**	**CV**
100	10	0.284	0.120	13	9.3	0.6	3	5.26	0.03
300	18	0.371	0.111	7	41.4	0.6	6	5.80	0.02

CV: Coefficient of variance.

**Table 3.** Comparison of published permeability values with the permeability of hydrogels exposed to 100 and 300 mg/dL glucose solutions, which resulted in 4.6% and 8.2% glucose-sensitive hydrogels, respectively.

**Material**	**Permeability (nm^2^)**
7.5% Agarose [21]	22.2
4% Agarose [22]	41.0
4.6% Glucose-Sensitive Hydrogel	52.6
8.2% Glucose-Sensitive Hydrogel	58.0
2% Agarose [21]	616.0
Human Vertebral Body (Transverse Direction) [26]	3.59 × 10^9^
